# In Silico Identification and Evaluation of Natural Products as Potential Tumor Necrosis Factor Function Inhibitors Using Advanced Enalos Asclepios KNIME Nodes

**DOI:** 10.3390/ijms221910220

**Published:** 2021-09-23

**Authors:** Dimitra Papadopoulou, Antonios Drakopoulos, Panagiotis Lagarias, Georgia Melagraki, George Kollias, Antreas Afantitis

**Affiliations:** 1 Biomedical Sciences Research Center “Alexander Fleming”, Institute for Bioinnovation, 16672 Vari, Greece; papadopoulou@fleming.gr; 2 Department of Physiology, Medical School, National and Kapodistrian University of Athens, 11527 Athens, Greece; 3 NovaMechanics Ltd., Nicosia 1070, Cyprus; drakopoulos@novamechanics.com (A.D.); lagarias@novamechanics.com (P.L.); 4 Division of Physical Sciences and Applications, Hellenic Military Academy, 16673 Vari, Greece; georgiamelagraki@gmail.com; 5 Center of New Biotechnologies & Precision Medicine, National and Kapodistrian University of Athens Medical School, 11527 Athens, Greece; 6 Joint Rheumatology Program, National and Kapodistrian University of Athens Medical School, 11527 Athens, Greece

**Keywords:** in silico, TNF, crystal structure, natural products, molecular dynamics, L929, TNF-TNFR1 binding, chemokines, Nepalensinol B

## Abstract

Tumor necrosis factor (TNF) is a regulator of several chronic inflammatory diseases, such as rheumatoid arthritis. Although anti-TNF biologics have been used in clinic, they render several drawbacks, such as patients’ progressive immunodeficiency and loss of response, high cost, and intravenous administration. In order to find new potential anti-TNF small molecule inhibitors, we employed an in silico approach, aiming to find natural products, analogs of Ampelopsin H, a compound that blocks the formation of TNF active trimer. Two out of nine commercially available compounds tested, Nepalensinol B and Miyabenol A, efficiently reduced TNF-induced cytotoxicity in L929 cells and production of chemokines in mice joints’ synovial fibroblasts, while Nepalensinol B also abolished TNF-TNFR1 binding in non-toxic concentrations. The binding mode of the compounds was further investigated by molecular dynamics and free energy calculation studies, using and advancing the Enalos Asclepios pipeline. Conclusively, we propose that Nepalensinol B, characterized by the lowest free energy of binding and by a higher number of hydrogen bonds with TNF, qualifies as a potential lead compound for TNF inhibitors’ drug development. Finally, the upgraded Enalos Asclepios pipeline can be used for improved identification of new therapeutics against TNF-mediated chronic inflammatory diseases, providing state-of-the-art insight on their binding mode.

## 1. Introduction

Chronic inflammatory disorders, such as rheumatoid arthritis (RA), Crohn’s disease, inflammatory bowel disease, and psoriasis, have been associated with deregulated inflammatory responses to multiple cytokines, which induce immune infiltration and fibroblast activation, leading to tissue damage [1]. Focusing on RA, according to recent recommendations [2], first-line treatment should include conventional disease-modifying antirheumatic drugs (DMARDs), such as methotrexate and glucocorticoids. For non-responsive patients, targeted synthetic DMARDs, inhibiting several kinases—Janus kinases (JAKs), mitogen-activated protein kinase (MAPK) and spleen tyrosine kinase (SYK)-Bruton’s tyrosine kinase (BTK) (SYK-BTK) [3] as well as biologic agents for blocking pro-inflammatory cytokines, including interleukine 1 (IL-1), interleukine 6 (IL-6) and tumor necrosis factor (TNF)—are administered.

TNF is a soluble cytokine, which belongs to the tumor necrosis family of homotrimeric proteins [4,5]. TNF functions through two receptors: TNFR1/p55 and TNFR2/p75. After the protein trimer has been formed, TNF binds to its receptors, leading, respectively, to the initiation of several inflammatory signaling pathways (e.g., NF-κB, JNK, p38-MAPK) [1]. Soluble TNF binds mainly to TNFR1, while transmembrane TNF binds to TNFR2. The monomeric and dimeric forms of TNF are inactive, and thus, in order to block the TNF relevant signaling, the inhibition of the trimeric form of TNF has been suggested [6].

Analyzing the TNF crystal structure, the inner part of each TNF monomer consists of hydrophobic residues, while the outer part constitutes the exposed surface of the cytokine. The top of the trimer presents polar interactions, with a possible salt bridge between Glu104 of one subunit and Arg103 of the other subunit. The center consists of hydrophobic contacts involving Tyr119, Leu57 and Leu157. Further hydrophobic interactions are present between Tyr59, Tyr119 and Tyr153 as well as Phe124 of an adjacent subunit. Finally, the bottom of the trimer contains a salt bridge between Lys11 of one subunit and the terminal carboxylate group of Leu156 of another subunit [5].

TNF has been broadly studied and reviewed in the literature [7,8,9,10,11,12,13], underlining the importance and the relevance of this growth factor as a valuable drug target.

As mentioned above, TNF antagonists have been widely used and proved effective in clinic [7]. However, these antagonists are mainly biologics, i.e., monoclonal antibodies (infliximab, adalimumab, certolizumab pegol, golimumab) [7,14,15] and fusion proteins (etanercept) [16], which bear certain drawbacks. Such disadvantages include causing hypersensitivity, increased risk of patients to develop serious infections, such as tuberculosis and hepatitis B because of the caused immune system suppression, loss of patients’ response during therapy due to arisen immunogenicity, and intravenous/subcutaneous administration as well as high cost of production and supply [8,10,11,17,18,19,20,21,22]. Thus, there is a great need for the development of potent small molecules that will efficiently inhibit TNF activity, as they can be inexpensively produced and distributed; they can be formulated to accommodate a plethora of administration routes (e.g., per os) while they can also present lower immunosuppressive side effects than biologics [8,10,11,21].

The first small molecule identified as a TNF inhibitor in vitro was suramin, synthesized in 1916 [23,24]. Hence, it has been used as a template for identifying other compounds with inhibitory activity, such as Evans blue and Trypan blue, while also setting the basis of structure–activity relationship (SAR) studies for this biomolecular target [25]. He et al. [6] developed SPD304, which exhibited a micromolar inhibitory activity in TNF-TNFR1 interaction, binding to the TNF dimer as revealed via X-ray crystallography, thus blocking the formation of the active TNF trimer. The structure elucidation of the bound ligand afforded valuable SAR information, which surprisingly does not include any hydrogen bond or electrostatic interactions of the bound ligand with the TNF dimer. All interactions were hydrophobic, while interactions of the ligand with TNF residues Tyr59 and Tyr119 were identified as highly important for TNF inhibition [5]. Starting from the ligand SPD304 in 2005, several small molecule TNF inhibitors have been developed, including heterocyclic compounds, small organic ligands, natural products (NPs) and metal-complexed compounds [10,11,12,26,27,28]. Blevitt et al. [29] discovered an inhibitor that forms an aggregating conglomerate, which antagonizes a protein subunit of the TNF trimer and alternates the quaternary structure of TNF upon binding. Thus, the formation of the TNF-TNFR1 is disrupted, due to the quaternary structure change induced by the aggregate. O’Connell et al. [27] and Dietrich et al. [12] developed small molecules with oral bioavailability that bind deeply in the TNF homotrimer, being able to allosterically stabilize an asymmetric complex or the trimer itself, leading to signaling inhibition. Xiao et al. [30] also proposed a compound that deforms the TNF trimer upon binding, leading to deregulated signaling upon binding of the TNF trimer to TNFR1.

In silico approaches were recently used to facilitate the identification of new potential anti-TNF therapeutics [21,31,32]. Computational modeling supports drug development, as it reduces both the time and the cost spent; it can predict clinical responses and it supports the 3R (replacement, reduction and refinement) concept requests [33]. Five novel TNF-α inhibitors were identified, following a structure-based virtual screening in combination with in vitro experiments [33,34]. Saddala and Huang [35] used a chemoinformatic workflow which employed pharmacophore modeling, docking and ADMET property prediction within the ZINC database to identify potential lead candidates with oral bioavailability for the inhibition of TNF; however, their biological activity has not been addressed. Recently, Melagraki et al. [36] developed two dual TNF-RANKL (receptor activator of nuclear factor κ-Β ligand) micromolar inhibitors, thus impeding the active biological form of both cytokines, which play a determinant role in chronic inflammatory diseases. Two compounds have been identified employing the Enalos [37] in silico workflow, which combines a structure-based chemical library screening approach together with a ligand-based consensus quantitative structure–activity relationship (QSAR) model filtering and molecular dynamics simulations in order to further investigate computationally the compounds’ binding mode [31,32,35]. Continuing their work in dual inhibitors, Melagraki et al. [9] proposed a NP, named A11 (Ampelopsin H), which efficiently blocks both TNF and RANKL, using a further automated version of the EnalosMD [9] workflow, which was recently included in the Enalos Asclepios KNIME nodes.

In this study, our aim is to identify further lead compounds that act as protein–protein interaction (PPI) inhibitors, binding to the inactive TNF dimer and inhibiting the formation of the active trimer. Development of small-molecule PPI inhibitors is very challenging [38], as a PPI inhibitor can act on certain “hot spots’’ on the protein surfaces [39,40,41], that are typically large, flat and solvent-exposed, while they are predominantly characterized by non-specific features (hydrophobic and electrostatic interactions) [42,43]. The large size and solvent exposure of the binding site require additionally that PPI inhibitors should achieve high potency and exhibit low molecular weight (MW), optimal lipophilicity and solubility [10,21,43]. To attain these pharmacokinetic properties of a prospective TNF small molecule ligand, we focused on NPs, deeming that, due to their natural origin as plant metabolites, they would bear an improved pharmacokinetic profile in comparison to synthetic compounds. Towards the identification of prospective PPI compounds with TNF inhibitory action in chemical libraries datasets, our group has further optimized the Enalos computational drug discovery pipeline. Conclusively, in this paper, we used our in silico pipeline to discover new NP lead compounds as potential TNF inhibitors, continuing our previous work in the field [9,36,44,45,46].

## 2. Results

### 2.1. Computer-Aided Drug Design (CADD)

#### 2.1.1. Initial Search and Filtering

Following up on our earlier efforts, we proceeded to an in silico optimization of compound A11 (Ampelopsin H) [9], using a modified version of our cheminformatics-aided workflow, EnalosMD and the Enalos+ suite [9,47,48,49]. More specifically, a data mining procedure was performed, using the Enalos PubChem similarity node [37,49], searching for NPs that would be analogs of Ampelopsin H. Molecular similarity (termed also as chemical similarity) suggests that structurally similar molecules are likely to have similar biological and physicochemical properties. This concept is widely known as the similarity principle, and it is fundamental in cheminformatics, as it is the basis of many property prediction models, as well as compound design models [50]. Our model uses a similarity method to search PubChem for molecules, which are similar to the structure of enquiry (Tanimoto similarity 85%) [51]. This search yielded 113 relevant compounds, which were selected for molecular docking simulations.

#### 2.1.2. Molecular Docking Simulations Using Enalos Asclepios KNIME rxDock Node

The molecular docking studies were performed using rDock [52] and the highest-score values were considered for the complexes’ construction. The docking was based on the TNF dimer complex with ligand SPD304 crystal structure (PDB code: 2AZ5) [6], after conducting a homology modeling to fill in missing residues (cf. Section 4). The docked SPD304 conformation reproduced its crystal form between the docked and the crystal SPD304 structure, while the docking score of SPD304 binding to TNF was calculated to be −54.269. After their preparation and their geometry optimization with the QM (quantum mechanics) node of the Enalos Asclepios workflow, the NPs that were identified by the similarity search were docked into the active site of TNF, and the highest-scored docking poses were selected. More specifically, the highest score of ampelopsin H was −45.140. The Enalos Asclepios KNIME nodes consist of a powerful tool, which fully automates the preparation of any ligand–protein complex and performs molecular dynamics simulations, offering optimal performance and versatility by employing a wide range of functionalities.

This refined docking step resulted in 53 compounds, which were deemed promising for further study. We acquired the 9 commercially available compounds out of these 53 identified compounds (Vitisin B, Miyabenol A, Kobophenol A, trans-Diptoindonesin B, trans-Miyabenol C, cis-Diptoindonesin B, cis-Miyabenol C, Nepalensinol B, Flexuosol A) to proceed to the phenotypic pharmacological testing and a more sophisticated in silico investigation.

### 2.2. Pharmacological Testing

**Figure 1:**
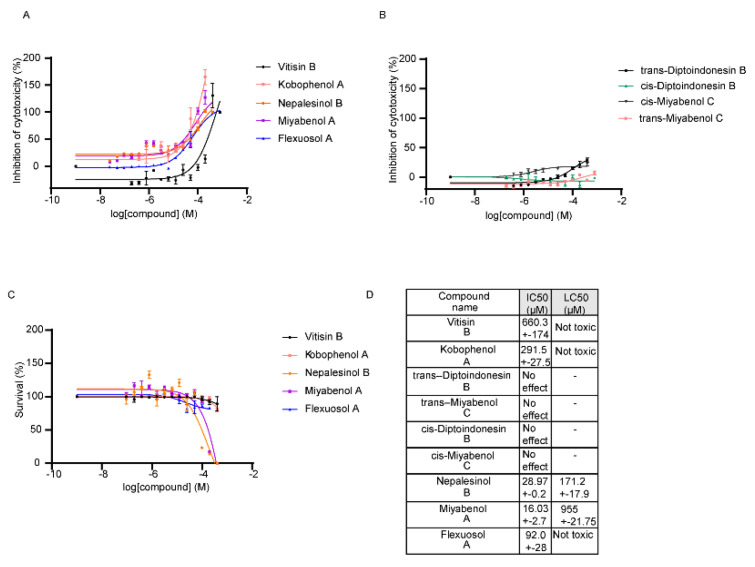
Ex vivo assays for the first pharmacological screening of the compounds. (**A**) Compounds that showed potential to downregulate the TNF induced cytotoxicity on L929 cells. (**B**) Compounds that were ineffective in downregulating the TNF induced cytotoxicity on L929 cells were excluded from further analysis. (**C**) Cytotoxicity of the effective compounds on L929 cells. All error bars indicate SEM values. (**D**) Conclusive table of the IC50 values of A and LC50 values of (**C**).

The pharmacological testing experiments included testing of the compounds in TNF-regulated assays ex vivo and in vitro. The first ex vivo screening of the compounds included quantification of the potential of the NPs to downregulate TNF-induced cytotoxicity in the L929 cell line, as previously reported [9,36]. Five out of the nine compounds tested showed an improved potential to block TNF activity (Figure 1A). Four compounds, named trans-Diptoindonesin B, trans-Miyabenol C, cis-Diptoindonesin B, cis-Miyabenol C, were excluded from further validation, as they seemed ineffective to block L929 TNF-induced death (Figure 1B). L929 cells were also used to assess the compounds’ toxicity, measured by a Crystal Violet assay, as described by Melagraki et al., 2017 (Figure 1C) [9,36]. The half maximal inhibitory concentration (IC50) values of the compounds’ efficacy in the L929 TNF-induced assay as well as the half maximal lethal concentration (LC50) values for the influence of the compounds in the L929 cell survival, are described in the table of Figure 1D. As for their toxicity, compounds Vitisin B, Kobophenol A and Flexuosol A reached a plateau of maximum cytotoxicity at >85% survival at a high concentration range, attaining a good therapeutic window (cf. Figure 1C). Nepalensinol B and Miyabenol A seemed the most effective compounds, as they supported L929 cells survival, upon TNF treatment, with an IC50 of 28.97 μM and 16.03 μM, while they presented LC50 in a higher concentration, at 171 μM and 955 μM, respectively.

**Figure 2:**
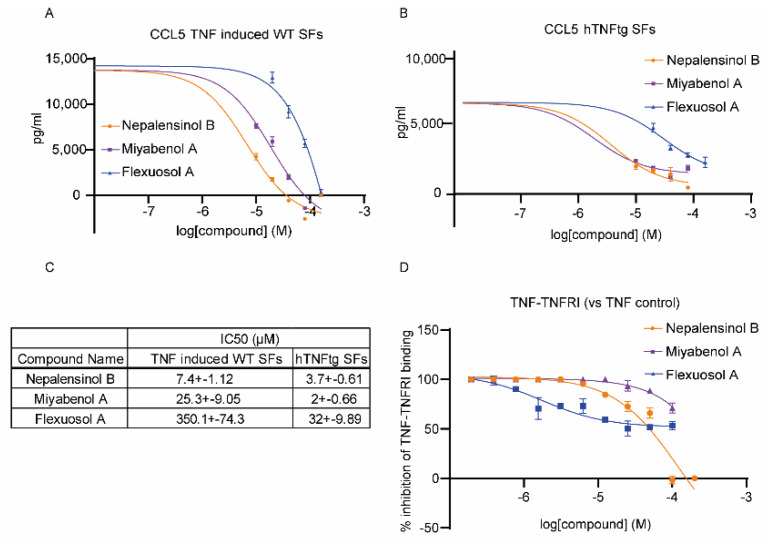
Validation of effective compounds. (**A**) Treatment of WT SFs with several concentrations of the compounds to assess the anti-inflammatory potential of the compounds on exogenous induction of TNF. (**B**) Treatment of hTNFtg SFs, which overexpress hTNF, with several concentrations of the compounds to assess the anti-inflammatory potential of the compounds. (**C**) Table with IC50s values of (**A**,**B**). (**D**) In vitro TNF-TNFR1 sandwich ELISA to assess the compounds’ potential to inhibit the interaction of TNF with its main receptor TNFR1. All error bars indicate SEM values.

Additionally, the anti-inflammatory potential of the top three compounds (Nepalensinol B, Miyabenol A and Flexuosol A) were studied in primary cells, using synovial fibroblasts (SFs), isolated either from the ankle joints of human TNF transgenic (hTNFtg) mice, or their wild-type littermates, as previously reported [53]. hTNFtg mice carry five copies of the hTNF transgene [54] and they spontaneously develop chronic polyarthritis, a disease similar to human RA; while they have been successfully used as drug testing platforms for anti-TNF therapeutics [55], SFs have been highlighted as the key driver cell type in this model of disease, as TNF/TNFR1 signaling in SFs is sufficient and necessary for the disease manifestation [56]. SFs were treated with different concentrations of the experimental compounds with (WT SFs, Figure 2A) or without (hTNFtg SFs, Figure 2B) the addition of hTNF. hTNFtg SFs secrete spontaneously hTNF, while wild-type (WT) SFs were stimulated with 10 ng/mL hTNF to increase their pro-inflammatory responses. At 48 h following the treatment of the SFs with different concentrations of the test compounds, their supernatants were analyzed by ELISA for the detection of CCL5/RANTES, a pathogenic chemokine in RA. Interestingly, Nepalensinol B and Miyabenol A were effective in downregulating CCL5, presenting, respectively, IC50s values of 7.39 μM and 25.25 μM in WT TNF-induced SFs and 3.7 μM and 2 μM in hTNFtg SFs. Flexuosol A, which presented a higher IC50 in the L929 TNF death–induced assay, was also about 10 times less effective in eliminating CCL5 levels in the tested concentrations.

Finally, the three aforementioned compounds were further tested for their potential to interrupt TNF-TNFR1 binding (Figure 2D). TNF/TNFR1 binding was quantified by an in vitro sandwich ELISA (as previously reported) [9,36]. Nepalensinol B showed the highest potential to interrupt TNF-TNFR1 binding, followed by Miyabenol A, with IC50 values of 90.2 ± 19 μM and 132 ± 14 μM, respectively (Figure 2C). However, Miyabenol A did not manage to interrupt the TNF-TNFR1 binding more than 70%, indicating that Nepalensinol B is probably a more promising inhibitor of the TNF-TNFR1 complex formation. Surprisingly, although Flexuosol A did not produce promising results ex vivo, it downregulated in vitro TNF-TNFR1 binding with an IC50 of 1.8 ± 0.45 μM at about 50%.

### 2.3. Molecular Dynamics and Free Energy Calculations

#### Molecular Dynamics Simulations with Enalos Asclepios KNIME MD-Simulation Node, Free Energy Calculations with MM-GBSA Method

**Figure 3:**
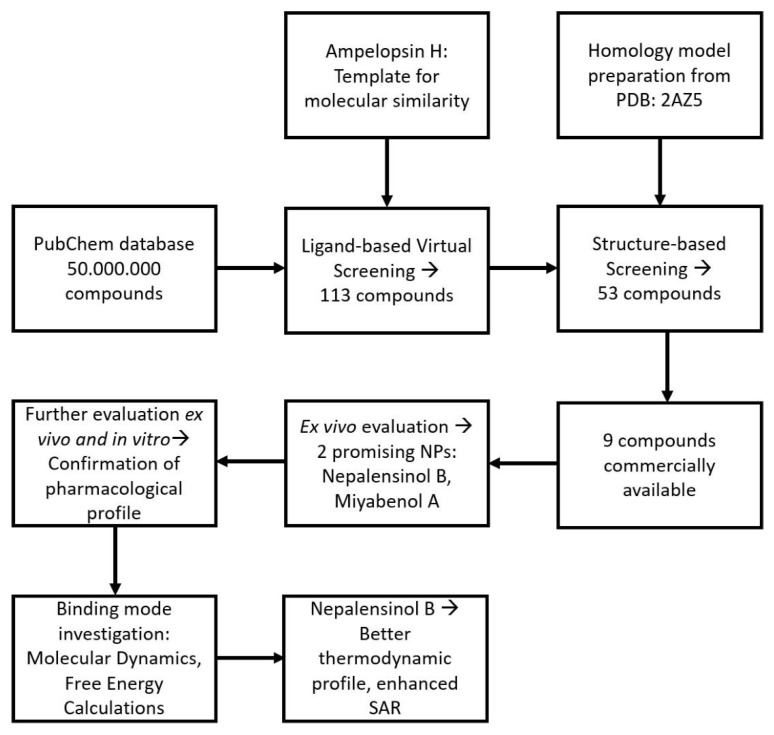
Schematic representation of the experimental workflow, containing a combination of pharmacological studies with computational models. The workflow starts by applying a molecular similarity search in PubChem, which yields 113 compounds. These compounds are further refined via a docking process in a homology model based on PDB: 2AZ5 (TNF dimer complex with ligand SPD304), resulting in 53 candidate ligands with a prospective affinity. Out of these compounds, 9 were commercially available, and thus they were acquired and further tested. An initial ex vivo screening in L929 cells verified that 2 compounds exhibit promising potency. Their optimal pharmacological profile was affirmed by further ex vivo testing in primary murine joints’ SFs and in vitro testing, evaluating the potential of the compounds to interrupt TNF binding to its main receptor, TNFR1. To investigate the compounds’ binding mode, a set of molecular dynamics simulations and free energy calculations was performed, suggesting that one of the two compounds, Nepalensinol B, could better serve as a candidate lead structure for anti-TNF drug design.

**Figure 4:**
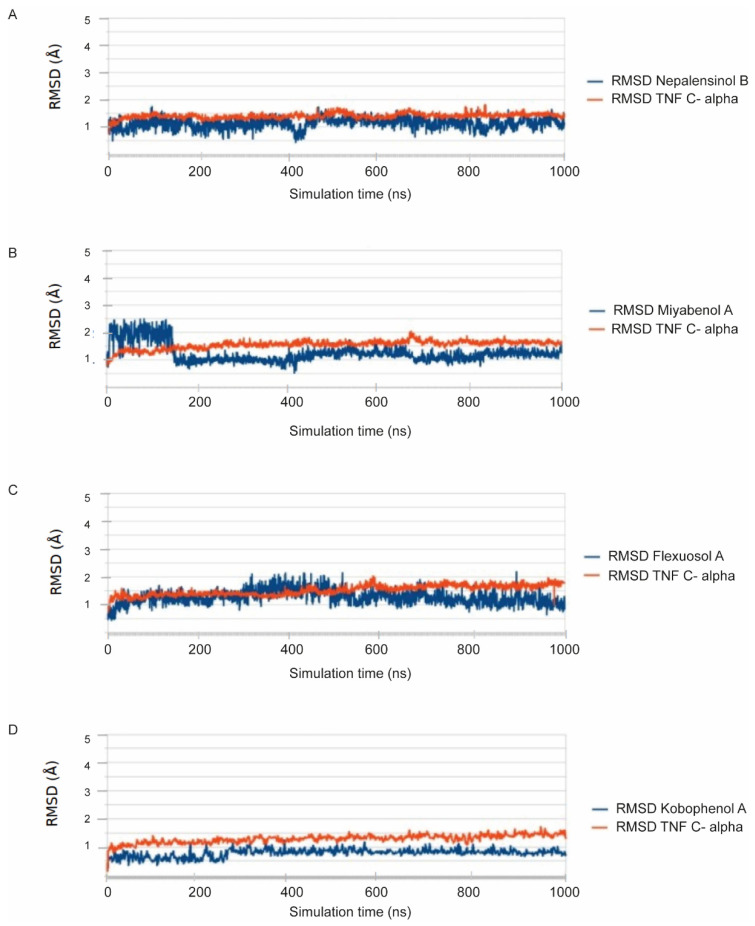
All-atom RMSD calculations. (**A**) Nepalensinol B; (**B**) Miyabenol A; (**C**) Flexuosol A and (**D**) Kobophenol A; in complexes with TNF.

Figure 3 summarizes schematically the entire workflow followed. Molecular dynamics (MD) simulations and free energy of binding calculations were conducted for the compounds exhibiting a pharmacological response to validate and enhance the predictive ability of our computational models, while investigating the binding mode of the compounds to TNF. The poses selected for complex formation and MD simulation, derived from the aforementioned docking procedure, in the classic merge of molecular docking and MD simulations. Each pose–receptor complex was embedded within TIP3P water solvent, and the 7 systems ran for 1 μs. According to the root-mean-square deviation (RMSD) graphs, the MD for all our ligands gave us stable trajectories (Figure 4). The analysis performed focused on the following aspects: (a) the number of the hydrogen bond and hydrophobic interactions that are formed between the cavity residues of TNF and the ligand compound, which are maintained until the end of the simulation for each ligand, (b) the conformational changes of the receptor after the simulation and (c) the binding affinity prediction and its most crucial terms.

Specifically, the MD results showed that protein structures stabilized early during the simulations in all complexes (cf. Figure 4). The RMSD values did not exceed 2.5–3 Å in TNF, and eventually all complexes showed convergence after ~350–400 ns (Figure 4). Cα-RMSD values for TNF complexes with the compounds were particularly stable, with an average deviation of 1.5–2.5 Å from the crystal structure (Figure 4). The ligand RMSD values for each TNF complex showed non-considerable changes throughout the trajectory. Miyabenol A, Nepalensinol B and Flexuosol A complexes showed RMSD 1.5–2.5 Å, with the most noteworthy fluctuation being observed in the first ~125 ns of the Miyabenol A–TNF simulation and after ~282 ns in the case of Flexuosol A–TNF simulation (Figure 4).

## 3. Discussion

**Figure 5:**
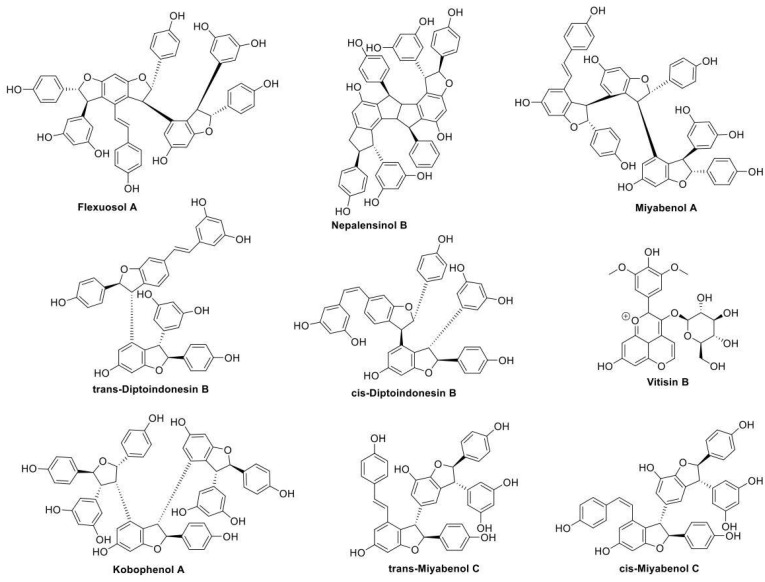
Commercially available compounds yielded from the cheminformatic screening procedure, searching for NPs similar to Ampelopsin H, that can potentially block the TNF trimer formation.

Interestingly, the vast majority of NPs (all except Vitisin B) yielded from the screening procedure are resveratrol oligomers, i.e., they belong to the chemical classes of stilbenes and stilbenoids (Figure 5).

These compound classes have been shown to possess a wide range of multifaceted activities of pharmacological interest, stretching from anticancer, antimicrobial, antioxidant, anti-inflammatory, antiplatelet, antidiabetic, hepato-, cardio- and neuroprotective, spasmolytic to tyrosinase inhibitory and ecdysteroid antagonist activities [57,58,59].

Examining every compound separately, Flexuosol A is a stilbenoid that belongs to resveratrol tetramers. It is isolated from the plants Vitis flexuosa Vitaceae and Dryobalanops lanceolata Dipterocarpaceae. It has exhibited moderate antibacterial activity against Staphylococcus strains [57,60]. Νepalensinol B is also a stilbenoid that belongs to resveratrol dimers and it can be found in the plants Kobresia Nepalensis Cyperaceae, Cenchrus Echinatus L. Poaceae and Sophora Stenophylla Fabaceae. Its total synthesis has been reported, albeit bearing an academic interest rather than providing a commercial perspective since it consists of a 13-step synthetic route. As for its pharmacological properties, it has shown anticancer activity by inhibiting DNA topoisomerase II (with a higher potency than etoposide) while exhibiting antiproliferative activity against adenocarcinoma cells [61,62,63]. Both cis-Miyabenol C and trans-Miyabenol C are resveratrol trimers. Cis-Miyabenol C is obtained by various Vitis, Muscadinia and Carex species; its pharmacological interest concerns inhibition of amyloid β fibril formation and ecdysteroid antagonist activity [58,64,65,66]. Trans-Miyabenol C is obtained by Caragana sinica of Leguminosae and various Vitis, Muscadinia and Carex species; it has been found to inhibit protein kinase C while presenting antioxidant activity [58,64,65,67,68]. Cis- and trans-Diptoindonesin B are stilbenoids, resveratrol trimers which were isolated from the plant Dryobalanops oblongifolia Dipterocarpaceae [69]. A recent in silico study suggested that they may act as sirtuine1 enzyme inhibitors, therefore demonstrating anticancer activity [70]. Additionally, Kobophenol A is a stilbene, resveratrol tetramer that has been isolated from various plants: Caragana sinica of Leguminosae [67], Carana sinica of Fabaceae [71], and many Carex Cyperaceae species (e.g., Carex kobomugi, Carex buchananii, Carex cuprina, and Carex folliculate) [58,72]. It has exhibited anti-inflammatory activity by downregulating the NF-κB signaling pathway that mediates inflammation [71]. In addition, Kobophenol A has yielded promising results as a potential prophylactic/therapeutic compound for osteoporosis and cardiotoxicity [73,74,75]. Furthermore, it has shown moderate antibacterial activity against Staphylococcus aureus [76] and antiproliferative activity [58]. A recent study reported that Kobophenol A could be a candidate lead compound against COVID−19 by inhibiting the interaction between angiotensin-converting enzyme 2 receptor of the host cells and the viral spike protein of SARS-CoV-2 [77]. Additionally, Miyabenol A is also a stilbene, resveratrol tetramer and it has been isolated from Vitis ficifolia Vitaceae and various Carex Cyperaceae species (e.g., Carex capillacea, Carex fedia var. miyabei, Carex hirta, Carex pendula, and Carex pumila) [58,78,79]. It has exhibited antimicrobial (Staphylococcus aureus and Bacillus subtilis), [58] anti-inflammatory [79], antioxidative and antiplatelet aggregation activity [80]. Vitisin B is a pyranoanthocyanine found in various red wines [81]. It has been successfully synthesized by reacting malvidine-3-monoglucoside with acetaldehyde [82] or vinyloxytrimethylsilane [83], and it has exhibited promising antioxidant activity [84].

Thus, the nine commercial compounds that emerged from our initial screening were, according to the literature, interesting candidates for further investigation, as their anti-inflammatory/antioxidant properties could be due to blockade of the TNF trimer formation.

Studying these nine compounds ex vivo in the L929 TNF induced necroptosis assay, [85,86], we found that, indeed, five compounds were able to support L929 cell survival upon addition to the cells. SPD304, when bound to TNF [6], was found to reduce L929 TNF–induced death (IC50 5 ± 0.2 μM), presenting however high toxicity (LC50 7.5 ± 0.2 μM) [9,87]. Notably, the new two best NPs identified, although ~5-fold less effective than SPD304, presented much less toxicity on the cells (in the range of two orders of magnitude). Additionally, mostly Nepalensinol B, and to a lesser extent, Miyabenol A, were able to downregulate chemokine ligand 5 (CCL5), which was found to be, among others, a pathogenic chemokine, being used for drug testing, both in human RA [88] and mouse chronic polyarthritis [89]. Both Nepalensinol B and Miyabenol A reduced CCL5 in both hTNFtg SFs, which spontaneously secrete high levels of chemokines, and wild-type (WT) SFs exogenously stimulated with human TNF in order to increase their pro-inflammatory potential. Using both cell settings, we show that compounds regulate the SFs responses by interfering with the TNF pathway itself. The combination of low toxicity along with the anti-inflammatory properties could assign to the most effective compounds, Nepalensinol B and Miyabenol A, a highly promising therapeutic potential, as they can serve as lead compounds addressing drug development for chronic inflammatory diseases. Miyabenol A and Nepalensinol B proceeded also to TNF-TNFR1–binding ELISA studies. Interruption of TNF-TNFR1 interaction contributes to the compounds’ desired properties, as disruption of TNF binding to its principal receptor, TNFR1, has been a long-desired goal in the development of novel therapeutics in chronic inflammatory models of disease [90]. Nepalensinol B interrupted TNF-TNFR1 binding at low concentrations devoid of cytotoxicity, indicating that this compound could be employed as a direct TNF inhibitor. Interestingly, Nepalensinol B completely abolished TNF-TNFR1 interaction at tested concentrations over 50 μM (Figure 2D), while Ampelopsin H achieved a downregulation of around 60% of TNF-TNFR1 binding in the same concentration range [9].

The docking studies investigating the binding affinity of the nine commercial compounds, using our recently developed TNF homology model, released through the Enalos Asclepios KNIME workflow (Supporting Information Figure S3), presented Nepalensinol B as a highly ranked ligand with a docking score of −35.722, although it was not the highest ranked. Interestingly, Miyabenol A did not yield a relatively high docking score (−23.104). On the contrary, Ampleopsin H was the highest ranked (second after SPD305 which was the reference compound) with a docking score value of −45.140, followed by Kobophenol A with −43.617, Vitisin B with −37.549, and trans-Diptoindonesin with −44.674. As these docking scores did not comply with the biological effect of the compounds, and in an effort to further investigate the binding and SAR of the ligands, we proceeded to conduct MD simulations. The elucidation and analysis of SAR afforded from the MD simulations led to a better understanding of the pharmacological profile of the compounds. Nepalensinol B’s docking pose exhibits interactions between the ligand and the carbonyl of the Gln52 amide group. Noteworthy hydrophobic pi–pi stacking interactions were observed between the two resorcinyl moieties of the ligand with their adjacent Tyr110 of each TNF subunit. The hydrogen bonds from visual inspection and distance analysis of the docking structure are formed between the backbone of amino acids Ser60, and Gln61 and the ligands’ phenolic hydroxyl group. This, in combination with the strong hydrophobic nature of the interactions that can be observed with the bulky phenol groups of the ligand, rises as the main driving force for the ligand’s core stabilization deep inside the cavity. These hydrophobic interactions are formed via the Leu57, Tyr57, Tyr119 and Tyr151 side chains.

**Figure 6:**
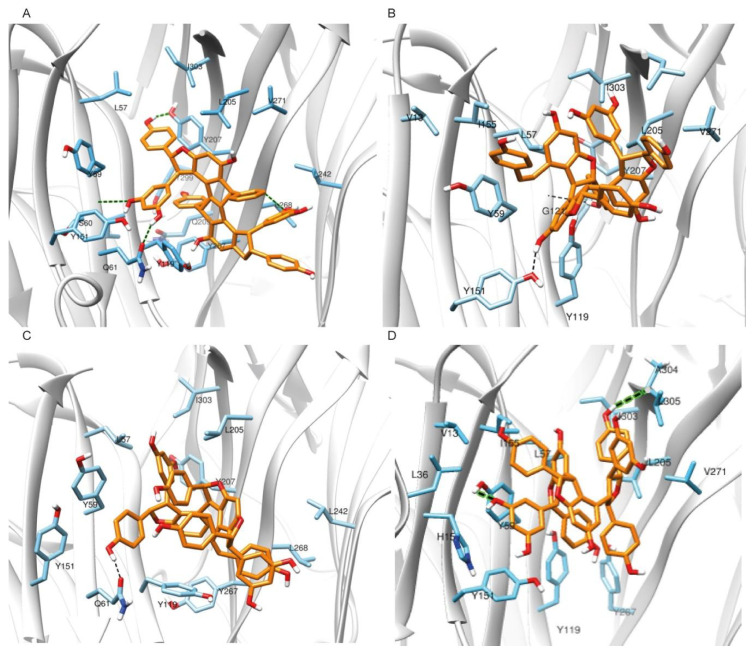
Main interactions between the compounds and the TNF binding cavity residues after 1000 ns MD simulation. Schematic representation of (**A**) Nepalensinol B; (**B**) Miyabenol A; (**C**) Flexuosol A and (**D**) Kobophenol A, when bound to the TNF cavity. Average conformations of the ligands within the cavity, along with protein residues that are involved in hydrogen bond interactions with the compounds are shown with black dashed lines, and green dashed for further clarification.

During the molecular dynamics simulation, enhanced van der Waals hydrophobic interactions are observed between the phenyl rings of Nepalensinol B and the TNF bulky hydrophobic residues of the cavity throughout the trajectory simulation (Figure 6A, Supporting Information Figure S1A), while the polar interactions with Ser60 and Gln61 identified in the docking pose are also present. After ~150–200 ns and until the end of the simulation, the Tyr110 pi–pi interactions are no longer present, but they preserve the hydrophobic environment, and they contribute indirectly to the further establishment of the hydrogen bond network, while also maintaining the local hydrophobicity. Additionally, interactions with the ligand and the side chains of Gln61, Gln209 and Tyr299, start gradually to be developed between the compound and the receptor. The strongest hydrogen bond interactions are observed after ~550 ns simulation, when the ligand changes orientation and enters even deeper inside the groove between the two dimers. There, Tyr299 tends towards the Gln209 side chain and opens space for the ligand to enter, facilitating interactions with the backbone of the latter. This change further stabilizes the interaction with the Tyr299 and Gly269 backbone and locks the ligand in this deeper position, causing further stabilization of the complex. The interactions described above, in combination with the hydrophobic interactions between the phenyl moieties of the ligand and the bulky phenyl groups of an array of tyrosines (Tyr59, Tyr119, Tyr299, Tyr207, Tyr267) as well as the aliphatic chains of Leu57, Ile 303 and Leu 205, act like a flexible hydrophobic horseshoe that covers the planar hydrophobic core of Nepalensinol B, providing the chemical environment for the ligand. Last but not least, the Tyr119 and Tyr267 also seem to further contribute to the ligand binding via pi–pi stacking interactions.

In summary, Nepalensinol B seems to develop a complex hydrogen bond interactions pattern, while simultaneously maintaining the hydrophobic van der Waals interactions with Leu305, Val271, and Ile155 and almost all the adjacent tyrosine residues. The hydrogen bond interactions network is developed between the hydroxyl groups of the ligand and Tyr151, Leu120 (backbone NH-) and Leu268 (backbone NH-group) of TNF. The latter can also further contribute to the Nepalensinol B binding stability via the isopropyl group of its side chain (Figure 6A, Supporting Information Figure S1A). The interaction pattern, which is formed especially after the second half of the 1μs MD, where the ligand slightly enters even deeper inside the cavity, suggests a strong affinity of Nepalensinol B, further refining the protein–ligand interactions. This provides an explanation of the ligand potency observed in our pharmacological data and mainly in the TNF-TNFR1 assay, despite its planar core.

For Miyabenol A, the main hydrogen bond interactions network can be observed between the ligand and the Tyr151 side chain hydroxyl group as well as the backbone amino group of Gly121 of TNF. Van der Waals hydrophobic interactions—especially pi–pi stacking—between the ligand and the Tyr59, Tyr119 and possibly the Tyr207 further contribute to the stability of the interactions between the ligand and the side chains of Leu57, Ile303, Leu205, Val271, Ile155 and Leu205 (Figure 6B, Supporting Information Figure S1B). Initially Gln61 amide forms hydrogen bonds towards the outer part of the cavity, but after ~200 ns of simulation, the ligand slightly shifts again towards the inner part of the cavity. As a result, the van der Waals hydrophobic network is enriched by additional interactions with the backbone amino groups of Gly121 and its adjacent Gly122. Even though Miyabenol A showed some relative instability during the first ~200–250 ns, by moving deeper inside the cavity, its hydrophobic interactions were eventually increased, due to gaining proximity towards Leu205, Ile303 and Leu57. Comparing the cores of Miyabenol A with Nepalensinol B, the former is much more flexible, thus allowing some significant mobility in the receptor’s cavity. As a result, the ligand moves further from the Gln61, a fact that does not seem to happen in the case of Nepalensinol B (Figure 6A), where the core is more rigid, and its dimensions fit much better within the cavity. In the case of Miyabenol A, the hydrogen bonds interaction network is less rich, but the core’s dimensions, despite its flexibility, allow optimal fitting inside the cavity, forming significant van der Waals interactions. Moreover, this flexibility allows Miyabenol A to form some noteworthy contacts with the Val13 side chain of TNF. Thus, the high potency of Miyabenol A can be attributed mainly to the formation of a rich hydrophobic network composed of strong and robust interactions. Thus, Miyabenol A can serve as an example of the importance of hydrophobic interactions in ligand binding; however, it can be argued that the two additional H bonds forming in Nepalensinol B exhibit their superiority when comparing the experimental TNF-TNFR1 binding results.

Flexuosol A, on the other hand, presents a less developed hydrogen bond interactions network, compared to Miyabenol A and Nepalensinol B (Figure 6D, Supporting Information Figure S2A). Compared to them, Flexuosol A’s core is the least planar. Nonetheless, the binding inside the cavity throughout the trajectory remains stable, due to the van der Waals hydrophobic interactions between the bulky Tyrosines of the binding cavity and the phenol groups of the ligand. In the docking pose and the initial steps of the MD trajectory, the main interactions are the one hydrogen bond between the phenolic fragments of Flexuosol A and the amide of Gln61 as well as the noteworthy hydrophobic interactions between the Leu205, Ile303 and the side chain of Leu57. The pi–pi stacking interactions between the ligand and Tyr59 are also possible, due to the latter’s adjacency.

However, the ligand does not seem to move deeper in the cavity as the simulation progresses, but it forms noteworthy interactions with Tyr151, Tyr59 and Tyr207 on the one side, and Tyr119 and Tyr267 on the other, causing some ligand’s packing, and thus, forcing it to create strong hydrogen bonds with the most adjacent Gln61. In addition, Flexuosol A does not seem to move deeper in the cavity, limiting its hydrophobic network to some interactions with the backbone carbonyl group of Leu268 and in a further proximity to some possible hydrophobic and polar interactions with Leu242 side chain and its backbone amino group, respectively (Figure 6C, Supporting Information Figure S2A). This binding profile provides a probable explanation for the inferred efficacy of Flexuosol A against TNF activation, despite the noteworthy binding stability throughout the trajectory. The ligand possibly can bind strongly, but it may not be enough to abolish TNF-TNFR1 binding, compared to Nepalensinol B.

Regarding Kobophenol A, the core structure of the ligand is much more flexible than Nepalensinol B. However, docking and molecular dynamics simulations showed that this is not enough for even more successful binding and potency. More specifically, this flexibility drives the ligand to adopt a more packed conformation within the cavity, forming hydrophobic interactions of a lower quality that prevent the formation of the interaction’s network observed in the cases of Miyabenol A or Nepalensinol B. However, some hydrogen bonds are formed between the resorcinyl group of the ligand and the Tyr59, Tyr267 as well as the backbone of Ala304. Although this hydrogen bond pattern with the above residues was also observed in Nepalensinol B (representing a fraction of its rich hydrogen bond network), the hydrogen bonds formed by Kobophenol A were weaker and not as robust throughout the simulation. Interestingly, the hydrogen bonds’ formation takes place after a close contact of Kobophenol A with His15, forming a pi–pi stacking interaction that provides an initial stabilization of the ligand, necessary for the latter to begin forming gradually the hydrogen interactions, starting with the Tyr59. Hydrophobic interactions are formed between the ligand and an array of bulky aliphatic side chains, including Leu36, Leu57, Val271, Ile303, Leu305, Ile155, Ile294 and Val13, but despite their number, they contribute only slightly to the hydrophobic stabilization of the ligand after ~750 ns of simulation. Secondary hydrophobic interactions, with even lesser effect, that seem to surround the ligands’ phenol moieties can be observed in Figure 6D, Supporting Information Figure S2B. It is, therefore, shown that the SAR of Kobophenol A is in agreement with its pharmacological profile, with a higher IC50 when compared to Miyabenol A, Nepalensinol B, and Flexuosol A in the L929 TNF-induced death assay.

In summary, only one significant hydrogen bond was identified between TNF and Flexuosol A; two hydrogen bond interactions were observed in the cases of Miyabenol A and Kobophenol A; and four in the case of Nepalensinol B. Interestingly, the compounds seem to bind slightly closer to the TNF subunit A than subunit B. The lack of extended hydrogen bond interactions network is noteworthy, and the hydrophobic character of binding was also experimentally observed in the case of SPD304 ligand [3]. In conclusion, the MD results confirmed the pharmacological experimental data, which highlighted Nepalensinol B and Miyabenol A as the most effective compounds, ex vivo and in vitro. The two ligands presented stable complexes when bound to TNF, adhering to both a more enhanced network of hydrophobic interactions and a higher number of hydrogen bonds than the other compounds (Figure 6).

**Figure 7:**
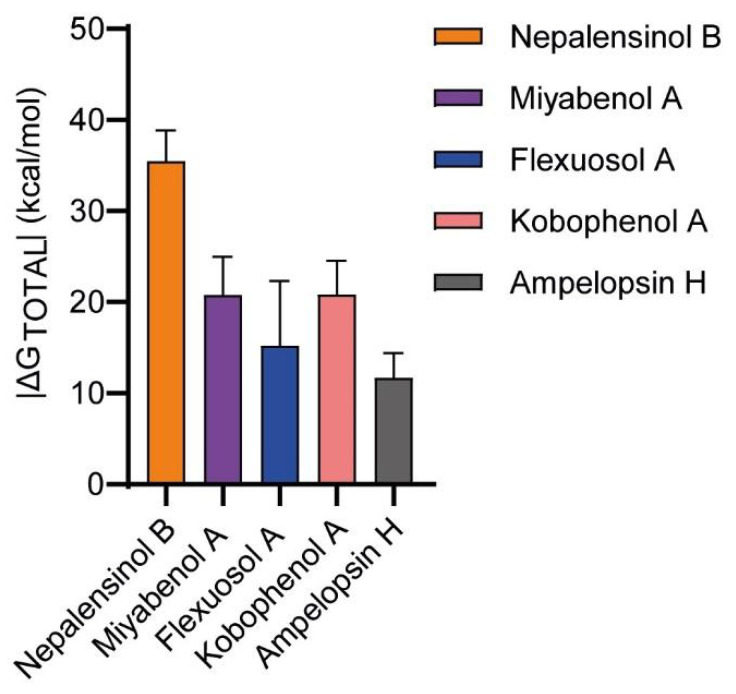
Histogram of the |ΔG total| values produced by MM-GBSA calculations. Nepalensinol Β is characterized by the best binding affinity (~−35 kcal/mol), 10 kcal/mol approximately higher than the other ligands. Noteworthy is the |ΔG| value of the Ampelopsin H, which is characterized by the lowest binding affinity.

As analyzed above, our model suggests that the stronger binding of Miyabenol A and Nepalensinol B could be explained by the respective SAR. However, a formidable lead compound should not only exhibit optimal SAR (which, ideally, have the potential to be further advanced in next generations of compounds), but should also render a promising binding energy profile based on prominent enthalpic interactions. Therefore, we performed a series of free energy calculations (MM-GBSA calculations Table 1; absolute free energy calculations, Figure 7) to further evaluate the binding thermodynamics of the aforementioned compounds.

Initially, free energy calculations were conducted fully automatically with the Enalos Asclepios KNIME workflow for each complex, employing MM-GBSA on the previously conducted 1 μs MD simulations (Table 1, Figure 7). In MM-PB/GBSA, we compute the difference between the free energies of the targeted state and a reference one, avoiding the energy calculation of the interactions between the solvent molecules. With this method, the free energy of binding (ΔG) is calculated, but the entropy has a large margin of error, causing significant uncertainty [91]. Moreover, in most cases, the entropy calculation is skipped to reduce the computational cost. This analysis suggested the higher potency of Nepalensinol B in comparison to Ampelopsin H since it demonstrated that it is characterized by a stronger and superior network of both polar (especially hydrogen bonds) and hydrophobic interactions, suggesting the better fitting and binding of the ligand within the cavity of TNF. This was not observed from molecular docking calculations, as can be depicted by their respective docking scores (Nepalensinol B: −35.722; Ampelopsin H: −45.140, Supporting Information Figure S3).

The decomposition of ΔG_total_, obtained from MM-GBSA in the terms presented on Table 1, suggests stronger van der Waals interactions (E_vdW_ = −62.44 kcal/mol) and noteworthy electrostatic/polar interactions (E_EL_ = −22.21 kcal/mol) for Nepalensinol B, with the specific term for the other ligands being considerably more positive. The aforementioned terms in combination with the highly negative ΔG_gas_ term implies the best fitting within the cavity, due to its planarity and the rich interaction network with the residues of the binding site, confirming the experimental data for possible potency, despite the high desolvation penalty (ΔG_solv_). Flexuosol A and Kobophenol A ligands exhibited a reduced binding affinity, compared to the others, in accordance with the experimental IC50 values. The ΔG_total_ decomposition into its terms showed average electrostatic and van der Waals energy for these ligands, but the desolvation penalty term was relatively high, causing this reduction in their estimated binding affinity. On the contrary, Miyabenol A and Ampelopsin H, which were characterized by a less broad polar interactions pattern but rich hydrophobic van der Waals network with a considerable number of bulky hydrophobic amino acids from the MD simulations, did not seem to confirm the experimental data, as their ΔG_total_ values were disproportionally more positive, compared to Nepalensinol B.

However, in the MM-GBSA free energy profiling of our ligands, we should consider both the fact that the level of SD (2.7–7.1 kcal/mol, cf. Table 1) is relatively high as well as the inherent limitations of the method, concerning its sensitivity. In addition, the big difference in the calculated ΔG of binding values (reaching to ΔΔG in the range of 10–15 kcal/mol) should yield a tremendously stronger binding, which, in turn, is expected to be depicted in the biological activity of the ligands. This does not happen, as the experimental IC50 values in the L929 TNF–induced death assay suggest a difference of 1–2 orders of magnitude between all the active ligands, which does not comply with such large differences in the free energy of binding. This becomes even more concerning since the compounds with highest and lowest |ΔG|, Nepalensinol B and Ampelopsin H experimentally are in the same IC50 range. In general, MM-PB/GBSA is a method most appropriate for free energy calculations in screening procedures, and such disadvantages are expected. Nonetheless, these results show a trend and provide insights to the thermodynamic profiling of ligand binding. The aforementioned methodological drawbacks can be addressed with more sensitive and accurate calculations of the absolute free energy of binding becoming feasible and being included in the computer-aided drug design (CADD) routine [92,93].

**Figure 8:**
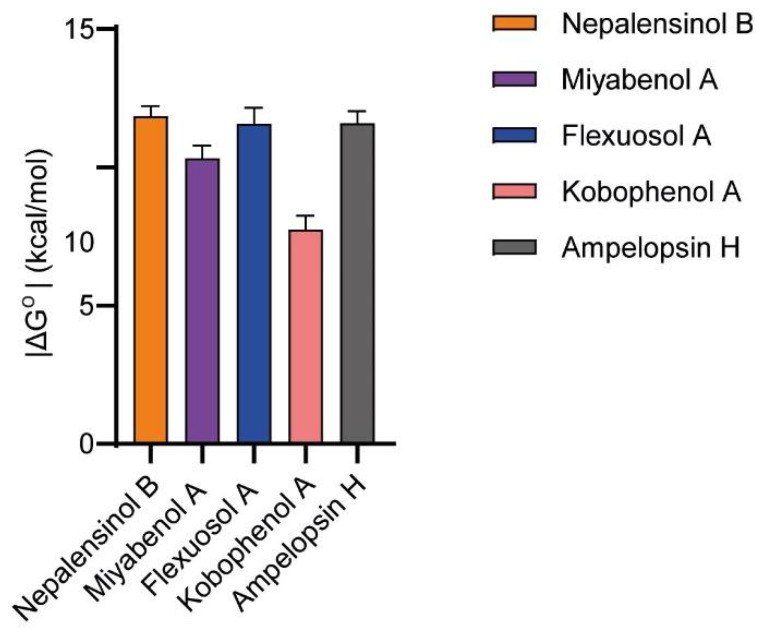
YANK-calculated values confirm the experimental observations for almost all ligands. Nepalensinol B remains the most active with the strongest estimated binding. Similar values are also observed in the case of Ampelopsin H. Miyabenol A is characterized by a moderate binding affinity, and finally, Kobophenol A shows the weakest binding. Flexuosol A shows a high binding affinity; however, its lower biological effect can be possibly due to further biological interactions or properties of the compound.

In an effort to further refine the thermodynamic profile of binding, the absolute binding free energies of the compounds were calculated, using the MBAR [94] method and the YANK 0.25.2 software [95]. For absolute free energy of binding (ΔG^o^) calculations, the method used applies several intermediate states between the initial and final states and simultaneously calculates the free energy differences between adjacent states. In this approach, several intermediate states are used between the initial and final states, and simultaneously calculates the free energy differences between adjacent states. The resulting final snapshots from the previously conducted MD simulations were selected as input for the ΔG^o^ calculations, and the respective values are presented in Figure 8. Comparing the results with the MM-GBSA histogram (Figure 7), we can observe that Nepalensinol B remains the best binding ligand (−11.851 ± 0.367) but there is a significant improvement in the results of Miyabenol A (−10.334 ± 0.465 kcal/mol) and Ampelopsin H (−11.591 ± 0.443). Kobophenol A remains the weakest binder (−7.754 ± 0.497), in accordance with the experimental results. Flexuosol A attributed a low ΔG^o^ (−11.582 ± 0.572) that supports its low in vitro IC50 in the TNF-TNFR1 assay. Its low biological effect ex vivo can be possibly due to properties of the compound that cause lowering of its efficacy in the cells, but not in the binding to its biological target (TNF) itself.

In the case of Miyabenol A (ΔG^o^ = −10.334 ± 0.465 kcal/mol), it can be observed that despite its relative core flexibility, it seems to develop not only some hydrogen bond interactions but also the richest hydrophobic network among all the ligands tested, as can be suggested from our MD simulations. The nature of its interactions could explain the observed increase in the estimated activity closer to the most potent ligands (Nepalensinol B and Ampelopsin H) and in agreement with the pharmacological results, compared to MM-GBSA, where we observe high deviation (Table 1). Similarly, focusing on Ampelopsin H, the hydrophobic interactions in combination with the rigid core that allows better fitting within the cavity support the improvement of the absolute binding calculation.

**Figure 9:**
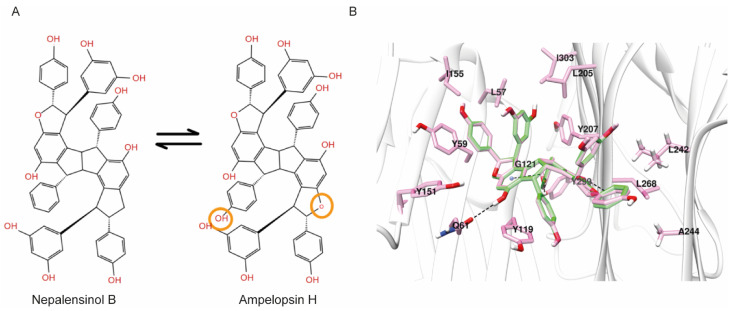
(**A**) The structures of Nepalensinol B and Ampelopsin H and their differences are highlighted with orange. (**B**) The binding of Ampelopsin after 1 μs MD and its transformation into Nepalensinol (green) for the absolute binding energy calculation.

Interestingly, the 2D structure of Nepalensinol Β and Ampelopsin H vary only in three atoms, as can be observed in Figure 9. A phenolic hydroxyl group and a furan oxygen atom are enough to convert Nepalensinol B into Ampelopsin H. YANK offers the possibility of ΔG^o^ calculation after mutations of the ligands, allowing us to compare the fitting of such similar compounds with different binding modes. Hence, after a 1 μs MD simulation, conversion of Nepalensinol B into Ampelopsin H was conducted in the final snapshot of the MD. The two mutations, i.e., the addition of the -OH group and the substitution of the methylene group into oxygen, were conducted to form Ampelopsin H, and the new complex underwent a ΔG^o^ calculation accompanied by an energy minimization and a number of short molecular dynamics to reach equilibration. The resulting extremely low absolute free energy of binding (ΔG^o^ = −2.471 ± 0.790) showed that the binding mode of Nepalensinol B would not be preferred by Ampelopsin H, possibly due to the polar -OH group presence that causes steric clash with the Tyr209, resulting in lack of free space within the cavity. Eventually, the hydroxyl group addition is abolished by the tyrosine, causing binding instability. Additionally, Ile303 can possibly contribute towards this instability, due to its adjacence to the hydroxyl group.

Surprisingly, the respective conversion of Ampelopsin H final snapshot pose into Nepalensinol B via mutating the oxygen of the furan ring to a methylene group and the -OH group with a −H, presented highly similar ΔG^o^ with Nepalensinol B after 1 μs MD simulation (−12.268 ± 0.534 and −11.851 ± 0.367, respectively). This suggests that Nepalensinol B may adopt both binding conformations within the cavity, with appropriate fitting in both cases. Possibly this is because its phenyl group can be adjacent to Leu242 and Ala244. This causes further stabilization and better fitting within the cavity via van der Waals interactions between the phenyl group and the side chains of these two residues. At the same time, all the remaining interactions are maintained, causing this slight tension to become an even more stable complex (Figure 9). Ile 155, Leu57, Ile 303 and Leu205 provide a hydrophobic environment for ligand binding. Additionally, the interactions include pi–pi of the phenol group with Tyr59, but also a hydrogen bond interaction between the phenol group of the ligand and the amide group of Gln61, the carbonyl group of the Gly121 backbone, and the amino group of the Leu268 backbone.

The hydrophobic core of Nepalensinol B, due to the lack of the oxygen atom, cannot create neither the hydrogen bond interactions between the compound’s core and the Leu268 NH group of the backbone, nor any water-mediated hydrogen bond. Hence, the absence of hydrogen bonds could prevent the ligand’s core to slide perpendicularly, and it could maximize the fitting via moving even deeper inside the groove between the two subunits, exploiting the free space of the Gly121 and Gly122. As a result, a new stronger van der Waals hydrophobic pattern and more pi–pi contacts are created, which contribute to the stabilization of the ligand. This stronger hydrophobic contribution is combined with the flexible phenol groups that create hydrophilic interactions of higher quality and lower energy—as expected from both the MM-GBSA and the YANK calculation—possibly due to their adjacence with the Gln61, Ser60, the Gly269 amino group of the backbone, the backbone of both Tyr299 and the Tyr207 (which also may contribute with a pi–pi stacking contact). Furthermore, we should note that Ampelopsin H is a symmetric molecule, a feature which facilitates it entering the binding pocket with the optimal direction to achieve the described SAR. Although Nepalensinol B lacks symmetry, it reverses this appearing disadvantage to an advantage since—as analyzed above—it can adopt both the binding mode of Ampelopsin H and another one of lower free energy, resulting in richer overall SAR.

As presented above, Nepalensinol B exhibits an excellent thermodynamic profile, achieving a significantly lower free energy of binding, predominately due to enthalpic parameters and low desolvation penalties. This fact, in combination with the advantageous SAR observed in docking and MD simulations, leading to a richer H bond network and a highly stable binding, renders Nepalensinol B a more promising lead compound for TNF drug discovery. Although both Nepalensinol B and Miyabenol A show efficient pharmacological properties combined with low toxicity, the former has a higher prospect as a lead structure since there are more features on the molecule that can be employed to further optimize next generations of ligand candidates.

## 4. Material and Methods

### 4.1. Cheminformatics Modeling—Computer Aided Drug Design (CADD)

#### 4.1.1. Enalos+ Similarity KNIME Node

Protocol/Parameters for Ampelopsin H similarity search.

Using Ampelopsin H as a reference compound within Enalos+ (Novamechanics, Nicosia, Cyprus), the Tanimoto similarity [51] was set at 85%, and a search was conducted in PubChem.

#### 4.1.2. Molecular Modeling

The homology model of the TNF receptor and the addition of missing TNF residues was conducted, using Modeller 9.16 [96]. The crystal structure 2AZ5 of the TNF dimer complex with SPD304 was utilized as the template for the homology modeling. All the protein complexes were prepared using PDBFixer (Stanford University, Stanford, CA, USA) [97]. The three-dimensional structures of the ligands were prepared by adding hydrogens, followed by energy minimization to obtain a low energy conformation necessary for the docking calculations, using the OpenBabel Toolbox (University of Pittsburgh, PA, USA) [98]. Hydrogen atoms were added also by OpenBabel, and the ligand’s geometry was further optimized using 6–31 Basis with GAMESS-US (Iowa State University, IA, USA) [99,100].

#### 4.1.3. Enalos Asclepios KNIME Workflow

**Figure 10:**
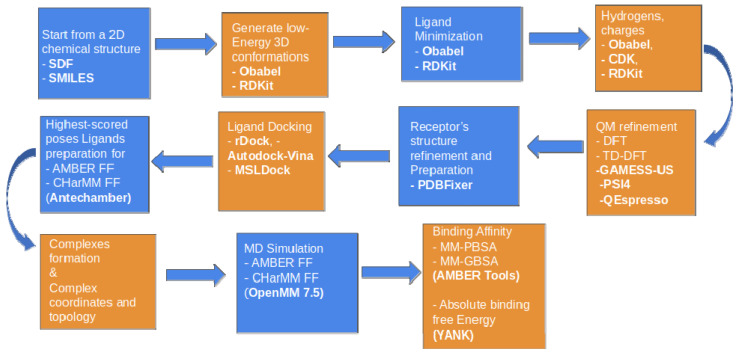
Enalos Asclepios KNIME workflow and its options for each node. A fully automated workflow for ligand-receptor preparation for docking, molecular dynamics simulations and binding affinity calculations.

Ligand minimization, hydrogen treatment, protonation, including the receptor preparation for docking, MD and MM-GBSA, were performed with our in-house developed Enalos Asclepios KNIME suite of programs (University of Konstanz, Zurich, Switzerland). Enalos Asclepios is an automated pipeline that includes all the major steps of ligand and receptor preparation docking, MD runs, and relative ΔG of the ligand binding: (a) initial model structures were constructed with AmberTools19 [101]. Missing TNF residues were added with PDBFixer [97]. The ff14SB force field [102] was used for the receptor, while the generalized AMBER force field (GAFF) [103] was used for the compounds’ representation. Geometry optimization and AM1-BCC [104] partial charges assignment for the ligands were conducted, using ANTECHAMBER [105]. The complexes’ topology and coordinate files were then run for 1000 ns, all-atom, unrestrained MD simulations with the GPU version of OpenMM 7.5 (Stanford University, Stanford, CA, USA) [106]. (b) Simulations were performed for (i) Miyabenol A–TNF, (ii) Nepalensinol B–TNF, (iii) Flexuosol A–TNF, (iv) Ampelopsin H–TNF, (v) Kobophenol A–TNF and (vi) Vitisin–TNF complexes in an explicit TIP3P water model [107] (Jorgensen et al., 1983) as the solvent at 300 K temperature, with the Langevin Thermostat, with the collision frequency set at 2.0 ps^−1^ [108], with 1 atm pressure with the GPU version of OpenMM. Periodic boundary conditions were applied with a 10 Å distance cutoff, using the particle mesh ewald (PME) method [109] for long-range interaction treatment. (c) Analysis of the results (RMSD, atomic fluctuations, and hydrogen bond calculations) was performed with the cpptraj version of AmberTools19 as part of the Enalos Asclepios workflow platform. The computational workflow, including its possible alternative options and settings, is represented schematically below (Figure 10).

#### 4.1.4. Molecular Docking with Enalos Asclepios KNIME Workflow

The Enalos Asclepios workflow of our method is presented in Figure 7, and it is further described in the following paragraphs. The ligand preparation nodes for docking and MD include a combination of MM and QM methods (DFT option was selected) for ligand geometry optimization to further refine the ligands before docking. The TNF receptor was prepared, protonated and hydrogen treated with PDBFixer, which was included in Asclepios as an automated part of the workflow. The same node was used also for filling the missing heavy atom. SPD304 was defined as a reference ligand. Docking calculations were conducted, using rDock (University of York, York, UK) molecular docking software [52] as part of workflow nodes. To define the active site of TNF the “reference ligand method” was employed with a radius of 6 Å around the bound SPD304; this area of the cavity was defined as the grid. The radius of the small and large probes used for mapping the TNF binding site was set to 1.5 Å and 4.0 Å, respectively. The integer for the maximum number of acceptable cavities was set to 1 with a minimum acceptable cavity volume of 100 Å^3^. A “cavity restrain function” with a weight of 1 was employed to prevent the ligand from exiting the docking site. Once the active site of TNF was defined and generated, each ligand was docked, using a 50 runs per ligand rDock job. The highest-scored docking solution of each ligand was selected, and the AMBER14SB force field option and ANTECHAMBER were used for the complexes’ formation as input for the MD simulations.

#### 4.1.5. Molecular Dynamics

Molecular dynamics simulations were performed with all-atom, unrestrained MD simulations with both the CPU and the GPU version of OpenMM 7.5 [106] of the Enalos Asclepios KNIME workflow node. The geometry optimization of the compounds was obtained with GAMESS-US [100] at the HF/6–31G*, using the DFT option of the QM refinement node of Enalos Asclepios workflow. AMBER topology and coordinates files were produced for each of the complexes for 1000 ns MD. The workflow included the following steps of the systems’ preparation, MD simulations and MD trajectory analysis procedures. Initial model structures were constructed with AmberTools19 [101]. The ff14SB force field [110] was used for the protein atoms, and the generalized AMBER force field (GAFF) [103] was used for parameterization of the SPD304, Miyabenol A, Flexuosol A and Nepalensinol B ligands. Geometry optimization and AM1-BCC partial charges [104] and formal charges were assigned by the ANTECHAMBER program. The AM1-BCC approach is based on a fast and effective parameterization method that reproduces particularly precise RESP charges [111]. Simulations were performed for each of the ligands in complex with the TNF homology model in the explicit TIP3P water model [107] solvent in a periodic boundary box of 10 Å cutoff. The particle mesh ewald (PME) method [109] was used for the long-range interactions treatment; periodic boundary conditions with a cutoff distance of 1 nm and a Langevin thermostat with collision frequency set at 2.0 ps^−1^ for the temperature regulation were used [108]. NPT ensemble, with a Monte Carlo barostat to keep constant pressure throughout the simulations, was used. The TNF complexes were neutralized by adding 4 Na^+^ counterions. Each minimization stage was carried out in 5000 cycles with a 1 nm cutoff. Hydrogen atoms were constrained using the SHAKE algorithm, and the AMBER14SB force field was selected for all of our complexes’ MD simulations. Equilibration and production runs were carried out, using the OpenMM 7.5 program. Our systems were minimized for 5000 steps, using the steepest descent method, followed by 5 ns equilibration with an NVT (constant particle number, volume, and temperature) setting. During the equilibration, the ligands were positionally restrained, whereas the proteins were allowed to relax. Restraints were removed for subsequent production runs that were carried out at 300 K and 1 atm pressure with an integration time step of 2 fs. CPPTRAJ [112] of AmberTools19 (University of California, San Francisco, CA, USA) was used for the data analysis of the MD output results: RMSD, atomic fluctuations, and hydrogen bond calculations. For the binding stability of our ligands within the TNF protein, for each snapshot, we then applied the MM-GBSA method as a method for more sensitive rescoring of our ligands’ binding after docking.

#### 4.1.6. MM-GBSA Method

The Molecular Mechanics Generalized Born (MM-GBSA) method can generally be used for the estimation of the relative ligand binding affinity and the calculation of the desolvation penalty. All analyses by MM/GBSA were implemented, using the MMPBSA.py [113] of AmberTools19, using the nodes of the Enalos Asclepios KNIME workflow. In the MM-GB/PBSA methods, the free energy (ΔG_bind_) between the ligand (L) and the receptor (R) form a complex L-R, which is calculated as follows:

L + R → L-R

The binding free energy can be decomposed into enthalpy and entropy contributions:

ΔG_bind_ = ΔH − TΔS (1)

The enthalpy (ΔH) is given by the following equation:

ΔH = ΔΕ_MM_ + ΔG_sol_ (2)

where ΔG_sol_ describes the change in the free energy of solvation due to ligand binding; ΔE_MM_ stands for the molecular mechanics energy (in gas phase), including the terms of bond stretching (ΔΕ_bond_), angle bending (ΔE_angle_), torsion rotation (Δε_tors_ ), van der Waals ΔE_vdW_ and electrostatic enegies (ΔΕ_elec_), as follows:

ΔE_MM_ = ΔE_bond_ + ΔE_angle_ + ΔE_tors_ + ΔE_vdw_ + ΔE_elec_ (3)

No cutoff was applied for the calculation of Δe_vdw_ and ΔE_elec_. Further decomposing the ΔG_bind_ for a ligand L and a receptor R to form the L-R complex, according to (3), we obtain finally Equation (4) as follows:

ΔG_bind_ = ΔE_bond_ + ΔE_angle_ + ΔE_tors_ + ΔE_vdw_ + ΔE_elec_ + ΔG_pol_ + ΔG_np_ − TΔS (4)

where the ΔG_pol_ term is the polar interaction, and ΔG_np_ stands for the non-polar contribution. ΔS is the system’s conformational entropy; the conformational entropy contribution to the binding free energy was neglected for computational cost reduction.

The MM-GBSA analysis was conducted for the 1400 last snapshots of the 1 μs MD simulation trajectories between 750 and 1000 ns. For MM/GBSA, the ΔG_pol_ term was calculated, using the atomic radii igb = 2 [114], and the salt molar concentration was set to 0.1 M. The ΔG_np_ term was determined based on the solvent accessible surface area (SASA), where the SURFTEN value was set to 0.005 kcal mol^−1^ Å^−2^. The solvent and solute dielectric constant values applied were 80.0 and 1.0, respectively.

#### 4.1.7. Absolute Binding Free Energies Calculation

For the binding free energy of a receptor (R)–ligand (L) complex (R-L), two calculations are conducted.

The first one is for the solvated complex and the second one is for the solvated ligand [115]. The ligand intramolecular contacts are kept, and there is an annihilation of the intermolecular ligand interactions to other molecules, provided that the temperature and pressure are steady.

R-L (solvated) → R (solvated) + L (gas) = ΔG^o^ _complex_ (5)

L (solvated) → L (gas) = ΔG^o^ _ligand_ (6)

R-L (solvated) → R (solvated) + L (solvated) = ΔG^o^ _binding_ = ΔG^o^ _complex_ − ΔG^o^ _ligand_ (7)

The (5) and (6) in combination give Equation (7). Our receptor–ligand complexes used as input for absolute free energy calculations are the final snapshots of the 1 μs MD simulations. These well-equilibrated structures were selected, and the free energy calculations and the analysis was conducted, using YANK v0.25.2 (University of Virginia, Charlottesville, VA, USA) [95,116], using its absolute binding calculation default protocol for runs with implicit solvent. The method combines the MD simulations with the absolute free energy calculations of the well-equilibrated structures. The last frame of the MD trajectory is used for ΔG_binding_ calculation with the Multistate Bennet Acceptance Ratio (MBAR)^116^ method. The strategy of the method includes some similarities to the well-established method for absolute free energy calculation method of MP-CAFEE. [117]

The λ = 0 state implies full interactions between the ligand and its surrounding atoms. The λ = 1 state implies the lack of these interactions. The free energy differences are calculated between the two states for the protein–ligand complexes (ΔG^o^ _complex_) and the ligand-only systems (ΔG^o^ _ligand_). In our case, 16 states between λ = 0 and λ = 1 were used in order to make the two states close enough, between which the free energy difference would be feasible to be calculated. For each of these states, the simulation was 1 ns. The input structures before the calculation were minimized for 500 iterations to further refine the receptor–ligand interactions, and 1000 iterations were selected with 500 steps for each iteration (steps 500,000 in total) for the simulations between each state. The parameters for the protein were the FF14SB force field and GAFF2 for ligand parameterization. The MD simulations for each state were conducted under the NVT ensemble at 300 K temperature; the charge method applied was BCC-AM1 and implicit solvent (OBC2 method) [114] was used for lower computational cost. No cutoff was selected as a non-bonded method. For the default harmonic restraints applied to keep the ligand near the protein, Langevin dynamics were run with a time step of 2 fs. We first switched off the electrostatic interactions (λ_electrostatics_ = 0 το λ_electrostatics_ = 1), and then the van der Waals (λ_sterics_ = 0 to λ_sterics_ = 1). We used 22 points: 11 λ_electrostatics_ (0.0, 0.1, 0.2, 0.3, 0.4, 0.5, 0.6, 0.7, 0.8, 0.9, 1.0) and 11 λ_steric_ (0.0, 0.1, 0.2, 0.3, 0.4, 0.5, 0.6, 0.7, 0.8, 0.9 and 1.0). Then, the free energy was then analyzed for each of all the trajectory snapshots via the (MBAR) [94] for the least biased free energy estimation between the two states.

### 4.2. Pharmacological Testing

#### 4.2.1. Cell Lines

The L929 cell line (NCTC clone) was obtained from ATCC (Manassas, VA, USA) and it was used for no more than 15 passages. The L929 TNF–induced cytotoxicity assay was performed as previously described [9,36].

In short, 3 × 10^4^ cells/well of cells were cultured on a 96-well plate, and next day compounds at different concentrations were added after being pre-incubated with 0.3 ng/mL recombinant human TNF-α (Peprotech, Rocky Hill, CT, USA) and 2 μg/mL Actinomycin D for 30 min at RT. The culturing medium alone, the medium with the respective Actinomycin D as well as the medium with both TNF and Actinomycin D were used as controls. After 18–22 h, supernatants were removed, and the plate was stained using a Crystal violet assay. The quantification was performed spectrophotometrically, and the values were calculated using the following equation:

(100* [(OD570 of sample) − (OD570 of TNF and ActD)])/((OD570 of ActD) − (OD570 of TNF and ActD))

For survival curves, 3 × 10^4^ cells/well of L929 cells were cultured for 24 h and treated with different concentrations of the compounds in medium. After 18–22 h, a crystal violet assay was performed, and the stained viable cells were measured, using a non-treated sample as a control.

#### 4.2.2. Mice

Primary mouse SFs were isolated from the ankle joints of mice with the indicated genotypes and cultured for three to four passages as previously described [53]. All mice were maintained in CBA;C57BL/6J genetic background under specific pathogen-free conditions in conventional, temperature-controlled, air-conditioned animal house facilities of BSRC Al. Fleming with 12 h light/12 h dark cycle was used, and the mice received food and water ad libitum.

#### 4.2.3. Chemokine Level Assay

For supernatants collection of SFs, cells were seeded at 2 × 10^4^ cells/ well of a 96-well plate in 1× DMEM (41966–029, Gibco, Thermo fischer Scientific, Austin, Texas, USA) supplemented with 100 U/mL Penicillin Streptomycin (P/S) and 10% Fetal Bovine Serum (FBS). The next day, the cells were starved in 1× DMEM, 0.5% FBS, 50 U/mL P/S, overnight (O/N). After O/N starvation, supernatants were gathered and stored in −80 °C. CBA/C57 WT cells were stimulated with hTNF 10 ng/mL (Peprotech, Rocky Hill, USA) or hTNF pre-incubated with different compound concentrations for 30 min. Supernatants were collected after 48 h of culture.

Supernatants were used for quantification of CCL5/ Rantes (DY478, DuoSet, R&D Systems, Minneapolis, MN, USA) by Elisa, which was performed according to the manufacturer’s protocol. 

#### 4.2.4. Natural Products

All NPs alphabetically named cis-Diptoindonesin B (CFN93706- Pubchem CID 643710), cis-Miyabenol C (CFN93332, Pubchem CID 5388319), Flexuosol A (CFN 93650, Pubchem CID 71308201), Kobophenol A (CFN92530, Pubchem CID 484758), Miyabenol A (CFN93701, Pubchem CID 16129868), Nepalensinol B (CFN93851, Pubchem CID 46890012), Vitisin B (CFN93805, Pubchem CID 74947464) and trans-diptoindonesin B (CFN93770, Pubchem CID 641676) and trans-Miyabenol C (CFN93716, Pubchem CID 6475924) were purchased from Wuhan ChemFaces Biochemical Co., Ltd., Wuhan, China.

#### 4.2.5. Statistical Analysis

Plotting, the estimation of the half maximal inhibitory concentration (IC50) and half maximal lethal concentration (LC50) values was performed, using GraphPad Prism 6 (GraphPad Software Inc., San Diego, CA, USA).

## 5. Conclusions

In summary, we have identified, experimentally studied and further elucidated the mode of action of new plant-derived TNF inhibitors, following up the work previously conducted by our groups [9]. These NPs block PPI by directly preventing the formation of the TNF trimer, while they exhibit low cytotoxicity levels and promising potency. The binding modes of the most efficient commercially available compounds were studied via docking and MD simulations, yielding valuable SAR information and providing insight into topological characteristics, which promoted the stronger binding of Nepalensinol B and Miyabenol A. The combination of MM-GBSA and absolute free energy of binding calculation also helped explain the stronger binding of Nepalensinol B and Miyabenol A, compared with the other less potent compounds tested. These compounds were further supported by either the strong hydrogen bond network (Nepalensinol B) or the rich van der Waals hydrophobic contacts (Miyabenol A) via the lower desolvation penalty and the lower ΔG, when compared with the other tested compounds. The MM-GBSA gave relatively poor correlation with the experimental results, the ΔG values differed disproportionately, and the standard deviation values were high. However, with the supplement of ΔG^o^ calculations, the results were significantly improved, providing an overall confirmation of the pharmacological data and also illustrating that Nepalensinol B can accommodate a second binding mode. Overall, Nepalensinol B and Miyabenol A were more potent in eliminating the TNF effect on L929 and primary murine joint fibroblasts, while the former interrupted more efficiently TNF-TNFR1 binding. Finally, the EnalosMD pipeline was advanced by adding the necessary modules to further refine our toolset; thus, it can successfully be used to identify new small molecules.

## Figures and Tables

**Table 1:** Energetic analysis for TNF in complex with Miyabenol A, Nepalensinol B, Flexuosol A, Kobophenol A and Ampelopsin H, as obtained by MM-GBSA calculations.

	Average kcal/mol	Std.Dev	Average kcal/mol	Std.Dev	Average kcal/mol	Std.Dev	Average kcal/mol	Std.Dev	Average kcal/mol	Std.Dev	Average kcal/mol	Std.Dev
	Miyabenol A		Nepalensinol B		Flexuosol A		Kobophenol A		Ampelopsin H		SPD304	
EvDW	−44.514	6.56	−62.4408	3.1846	−31.391	6.848	−46.87	3.6866	−45.9798	3.461	−43.9821	2.5799
EEL	−15.963	6.307	−22.2184	5.5841	−19.287	10.104	−18.6796	6.4461	−13.0541	4.7604	−111.752	5.2156
EGB	45.629	6.965	56.5623	4.9438	39.683	9.983	50.9639	6.0277	45.0963	6.6032	144.1032	5.9402
ΔGGAS	−60.484	9.413	−84.712	6.0403	−50.661	11.583	−65.5466	7.5489	−59.0364	6.6642	−155.734	6.5496
ΔGSOLV	39.698	6.587	49.2238	4.8589	35.445	9.584	44.7208	5.8006	39.3336	6.47	139.36	5.8403
ΔGTOTAL	−20.786	4.184	−35.4882	3.3524	−15.216	7.09	−20.8258	3.7012	−19.7027	2.6903	−16.3744	2.8002

## Data Availability

Data sharing not applicable. No new data were created or analyzed in this study. Data sharing is not applicable to this article.

## Supplementary Materials

The following are available online at https://www.mdpi.com/article/10.3390/ijms221910220/s1.
