# Supplementary material for: In Silico Identification and Evaluation of Natural Products as Potential Tumor Necrosis Factor Function Inhibitors Using Advanced Enalos Asclepios KNIME Nodes
Source: Int J Mol Sci. 2021 Sep 23;22(19):10220. doi: 10.3390/ijms221910220 (PMC8508374; doi:10.3390/ijms221910220)
Supplement: Supplementary file 1 [file ijms-22-10220-s001.zip › ijms-1329425-supplementary.pdf]

Supplementary

In Silico Identification and Evaluation of Natural Products as Potential Tumor Necrosis Factor Function Inhibitors Using Advanced Enalos Asclepios KNIME Nodes

Dimitra Papadopoulou [1,2], Antonios Drakopoulos [3], Panagiotis Lagarias [3], Georgia Melagraki [4], George Kollias [1,2,5,6,*] and Antreas Afantitis [3,*]

[1] Biomedical Sciences Research Center "Alexander Fleming", Institute for Bioinnovation, 16672 Vari, Greece; papadopoulou@fleming.gr

[2] Department of Physiology, Medical School, National and Kapodistrian University of Athens, 11527 Athens, Greece

[3] NovaMechanics Ltd., Nicosia 1070, Cyprus; drakopoulos@novamechanics.com (A.D.); lagarias@novamechanics.com (P.L.)

[4] Division of Physical Sciences and Applications, Hellenic Military Academy, 16673 Vari, Greece; georgiamelagraki@gmail.com

[5] Center of New Biotechnologies & Precision Medicine, National and Kapodistrian University of Athens Medical School, 11527 Athens, Greece

[6] Joint Rheumatology Program, National and Kapodistrian University of Athens Medical School, 11527 Athens, Greece

* Correspondence: kollias@fleming.gr (G.K.); afantitis@novamechanics.com (A.A.)

A

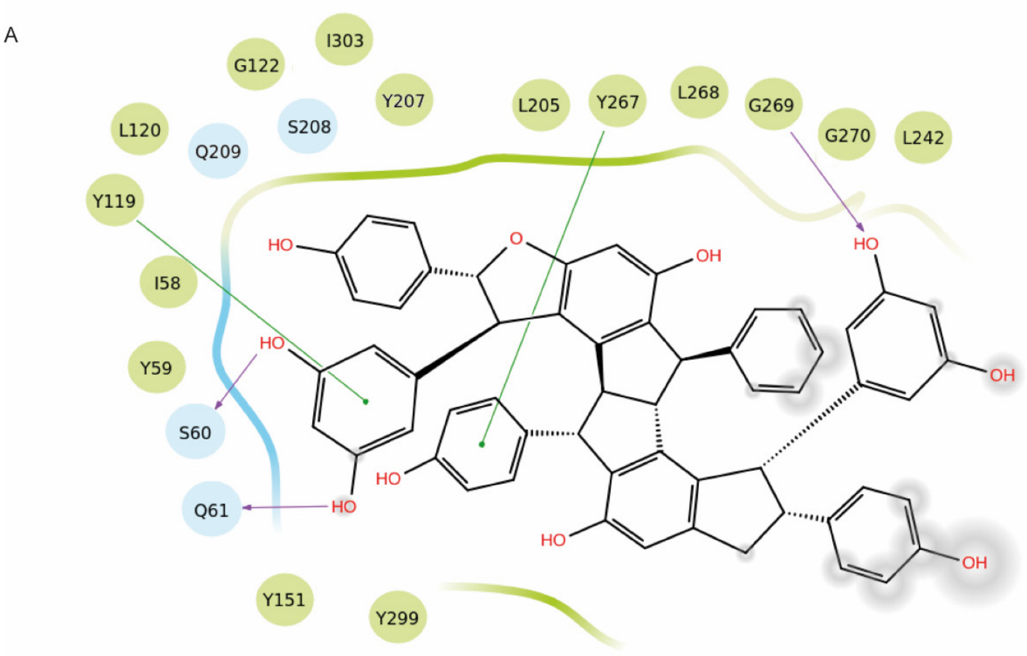

B

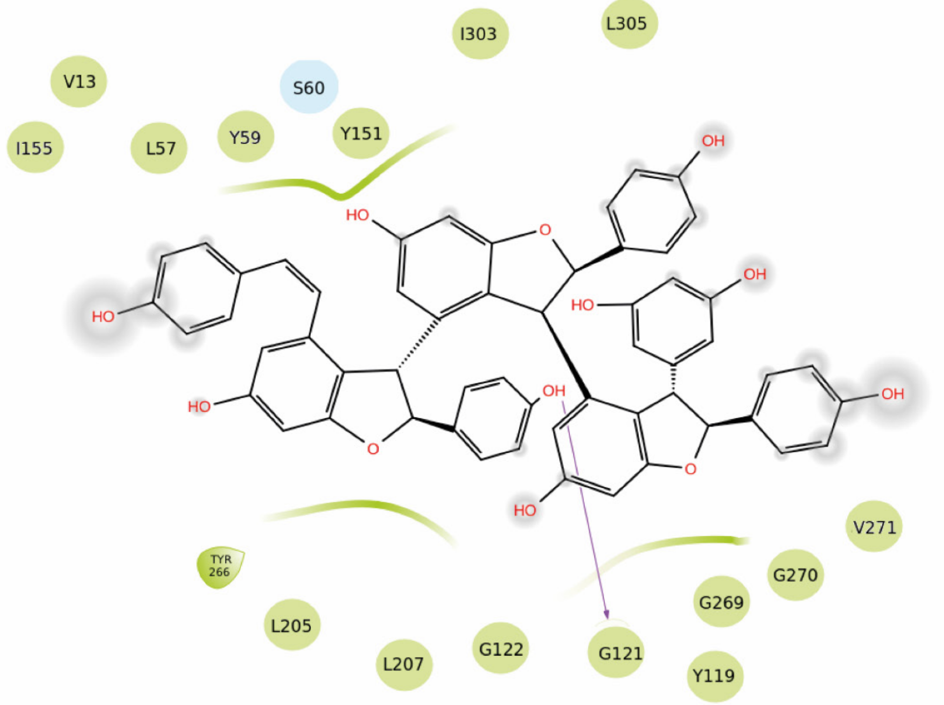

Supplementary Figure S1. SAR in 2D A. Nepalensinol B, B. Miyabenol A.

A

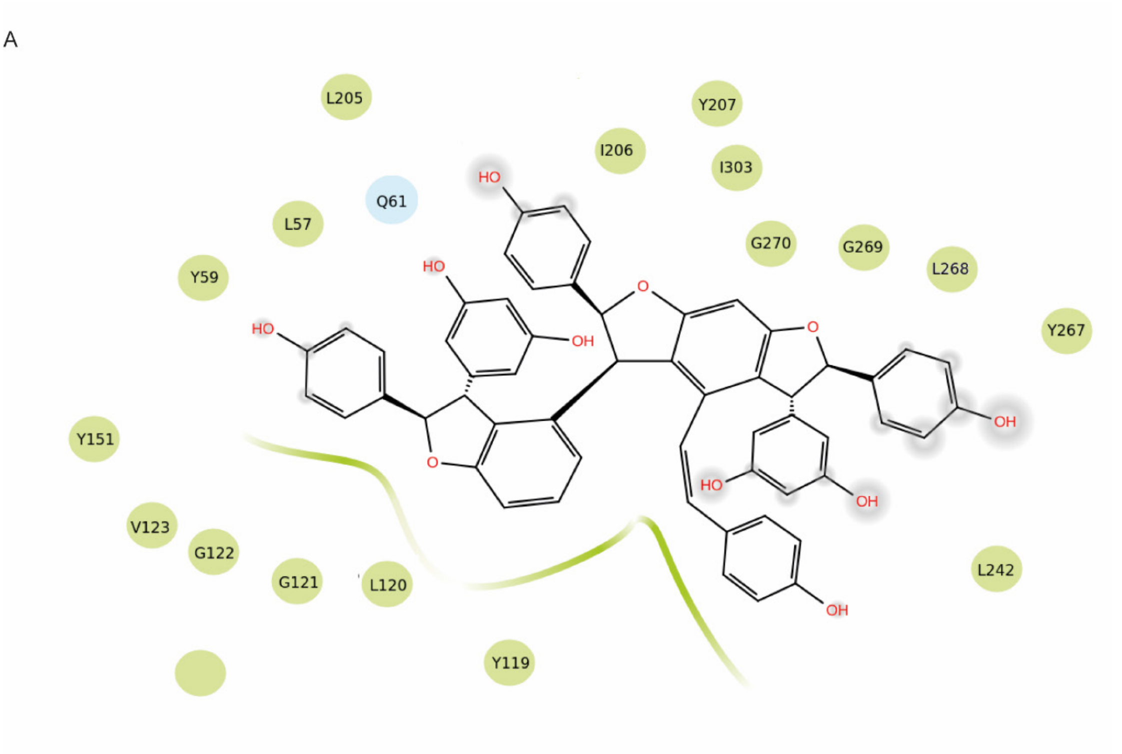

B

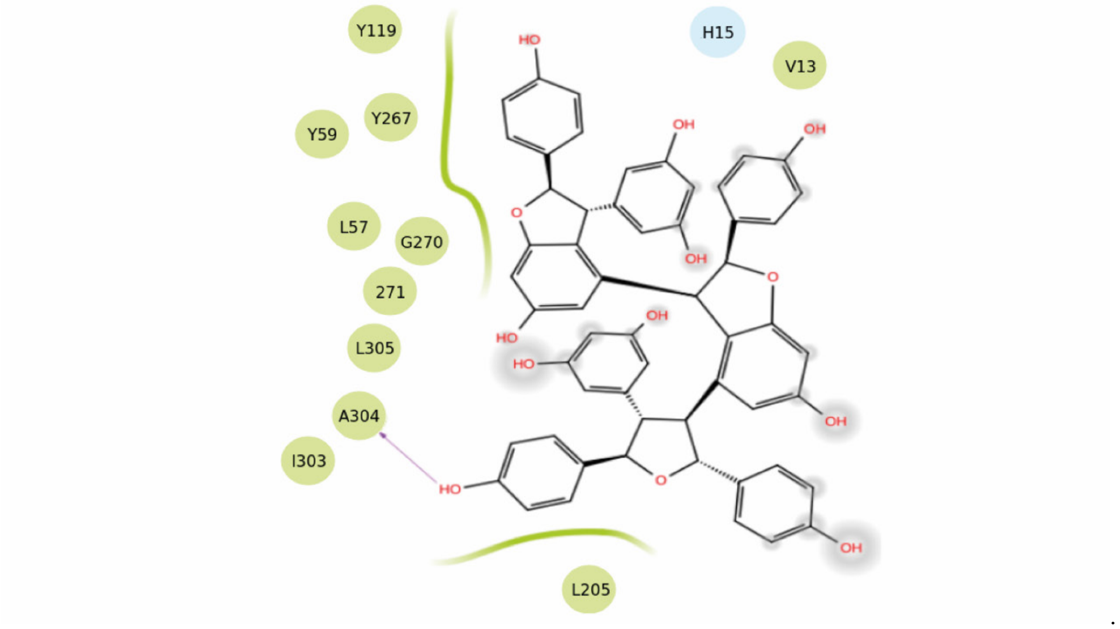

Supplementary Figure S2. SAR in 2D A. Flexuosol A, B. Kobophenol A

**Ligands Name**	**Docking Scoore (rDock)**
Ampelopsin H	-45.14
SPD304	-54.269
Nepalensinol B	-35.722
Miyabenol A	-23.104
Flexuosol A	-32.603
Kobophenol A	-43.617
Vitisin B	-37.549
trans-Diptoindonesin B	-44.674

Supplementary Figure S3. Docking scores of compounds
